# Calreticulin Ins5 and Del52 mutations impair unfolded protein and oxidative stress responses in K562 cells expressing CALR mutants

**DOI:** 10.1038/s41598-019-46843-z

**Published:** 2019-07-22

**Authors:** Simona Salati, Elena Genovese, Chiara Carretta, Roberta Zini, Niccolò Bartalucci, Zelia Prudente, Valentina Pennucci, Samantha Ruberti, Chiara Rossi, Sebastiano Rontauroli, Elena Enzo, Laura Calabresi, Manjola Balliu, Carmela Mannarelli, Elisa Bianchi, Paola Guglielmelli, Enrico Tagliafico, Alessandro M. Vannucchi, Rossella Manfredini

**Affiliations:** 1 Centre for Regenerative Medicine “Stefano Ferrari”, Department of Life Sciences, University of Modena and Reggio Emilia, Modena, Italy; 2 Institute for Cell and Gene Therapy & Center for Chronic Immunodeficiency, University of Freiburg, Freiburg, Germany; 3 CRIMM, Center for Research and Innovation for Myeloproliferative Neoplasms, Department of Experimental and Clinical Medicine, AOU Careggi, University of Florence, Florence, Italy; 4 Center for Genome Research, University of Modena and Reggio Emilia, Modena, Italy; 5 Department of Medical and Surgical Sciences, University of Modena and Reggio Emilia, Modena, Italy

**Keywords:** Myeloproliferative disease, Stress signalling

## Abstract

Somatic mutations of calreticulin (CALR) have been described in approximately 60–80% of JAK2 and MPL unmutated Essential Thrombocythemia and Primary Myelofibrosis patients. CALR is an endoplasmic reticulum (ER) chaperone responsible for proper protein folding and calcium retention. Recent data demonstrated that the TPO receptor (MPL) is essential for the development of CALR mutant-driven Myeloproliferative Neoplasms (MPNs). However, the precise mechanism of action of CALR mutants haven’t been fully unraveled. In this study, we showed that CALR mutants impair the ability to respond to the ER stress and reduce the activation of the pro-apoptotic pathway of the unfolded protein response (UPR). Moreover, our data demonstrated that CALR mutations induce increased sensitivity to oxidative stress, leading to increase oxidative DNA damage. We finally demonstrated that the downmodulation of OXR1 in CALR-mutated cells could be one of the molecular mechanisms responsible for the increased sensitivity to oxidative stress mediated by mutant CALR. Altogether, our data identify novel mechanisms collaborating with MPL activation in CALR-mediated cellular transformation. CALR mutants negatively impact on the capability of cells to respond to oxidative stress leading to genomic instability and on the ability to react to ER stress, causing resistance to UPR-induced apoptosis.

## Introduction

Calreticulin is a major Endoplasmic Reticulum (ER) chaperone playing multiple roles in a large variety of cell processes, such as quality control of protein folding^1^ and Ca_2_ + homeostasis^2^. CALR has also been found in the cytoplasm, at the cell membrane and in the extracellular matrix, where it takes part in many physiological processes, including cellular stress responses^3^.

CALR consists of three distinct structural and functional domains: the N-terminal domain and the proline rich P-domain play a role in chaperone activity, while the C-terminal domain is responsible for Ca2+ homeostasis. The C-terminal domain also contains a KDEL sequence for retrieval/retention in the ER.

In 2013, two groups reported the discovery of mutations in the CALR gene in Myeloproliferative Neoplasms (MPNs), particularly in 60–80% of JAK2 and MPL unmutated Essential Thrombocythemia (ET) and Primary Myelofibrosis (PMF) patients^4,5^.

Mutations are located in exon 9, consist of deletions and/or insertions and result in a mutant protein with a novel C-terminal. CALR mutations cause the loss of most of the acidic C-terminal domain and the KDEL signal, which might lead to protein mislocalization and aberrant protein function and stability. The mutation has been detected in hematopoietic stem and progenitor cells and hierarchical clonal analyses showed early acquisition of the CALR mutation, consistent with its role as initiating event of the disease.

Recently, Marty *et al*.^6^ showed that CALR mutations are able to induce the development of an ET-like phenotype in mice, whilst Chachoua *et al*. demonstrated the requirement of the Thrombopoietin receptor (MPL) for CALR-mediated cellular transformation^7^. Finally, two research groups have shown the physical interaction of CALR mutants and MPL; in this scheme, the N-terminal domain of CALR mutated protein is able to interact with the extracellular domain of MPL causing its activation^8,9^.

Nonetheless, the role of CALR mutations in the development of MPN has been only partially elucidated, since no data are available so far on the effects exerted by these mutations on the physiological functions that CALR plays in the ER. As described above, CALR exerts its major functions in the ER, by regulating processes tightly interconnected such as protein folding and response to oxidative stress^1^. To date, no report has described whether and how CALR mutants could affect unfolded protein response (UPR) and oxidative stress response, two processes whose alteration could make the cell resistant to ER stress-induced apoptosis and prone to the accumulation of mutations leading to genomic instability.

It has already been demonstrated that calreticulin plays a pivotal role in the quality control of protein folding^1^. Moreover, CALR overexpression has been shown to increase cell sensitivity to H2O2-induced cytotoxicity^10^, indicating that CALR plays a critical role in oxidative stress–induced apoptosis.

Since CALR has been demonstrated to be involved in ER and oxidative stress response in different cell types^11^, here we investigated whether CALR mutations could be responsible for an abnormal response to ER and/or oxidative stress and whether this aberrant response could represent a pathogenetic mechanism cooperating with MPL activation in CALR-mediated cellular transformation.

Our results demonstrated that CALR mutations are able to impair both the UPR and DNA repair upon ER and oxidative stress respectively. Altogether, these data suggest a novel role for CALR mutations in MPN pathogenesis, involving resistance to ER stress-induced apoptosis and reduced capability to repair oxidative DNA damage.

## Results

### Gene expression profile evidences the downregulation of ER stress response and oxidative stress genes in CALR mutated cells

In order to unravel MPL-independent mechanisms underlying the effect of CALR mutations on MPN pathogenesis, we analysed the transcriptional changes induced by the *CALR*ins5 or *CALR*del52 overexpression in K562 cells, which lack MPL expression^12,13^.

In a set of three independent experiments, K562 cells were transduced with the LCALRwtIDN (CALR wild-type, CALRwt), LCALRdel52IDN (CALRdel52), LCALRins5IDN (CALRins5) retroviral vectors. After NGFR-based purification, the transduced cells were profiled by means of Affymetrix U219 Array.

**Figure 1:**
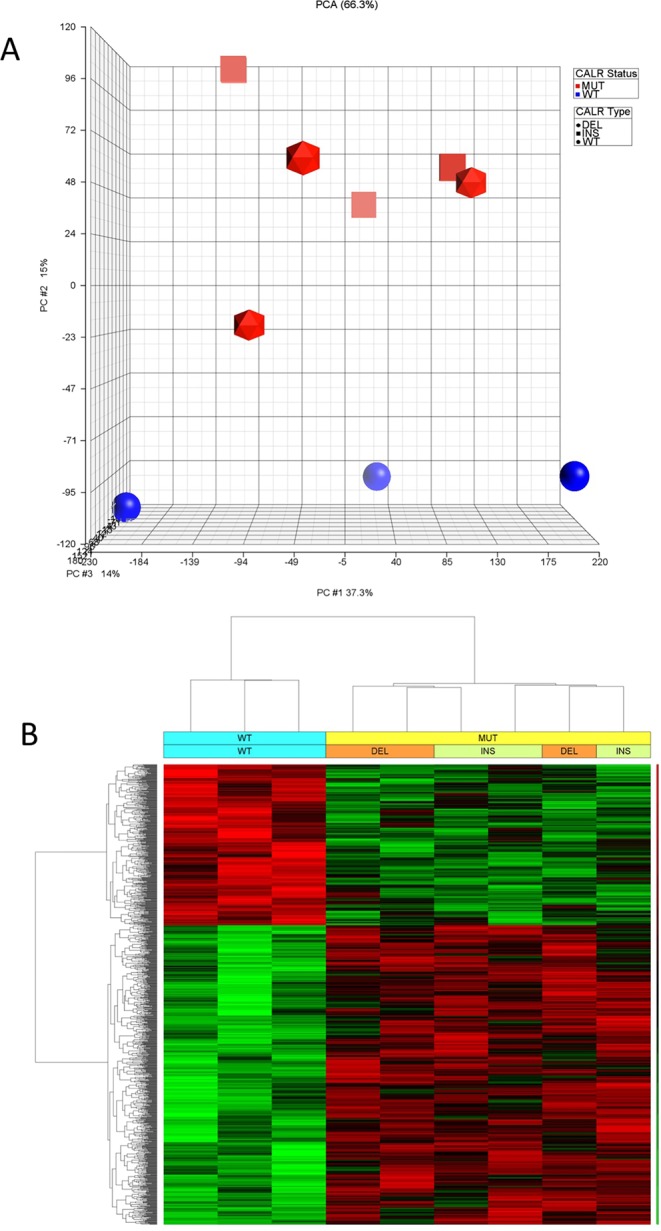
Gene expression profiling data for *CALR*wt, *CALR*del52 and *CALR*ins5 K562 cells. (**A**) The Principal component analysis was performed by using the PCA module implemented in Partek GS. wt-transduced K562 are shown in blue, while CALRdel52 and CALRins5-transduced K562 are shown in red. Mutational status (i.e.wt, CALRdel52 and CALRins5) is indicated by using different shapes. (**B**) Hierarchical clustering and heat map analysis for the transcripts differentially expressed in both CALRdel52 and CALRins5-transduced cells vs wt-transduced cells using a gene list having an unadjusted p-value <0.01 in the ANOVA analysis. For each transcript, red bars indicate relatively high signal intensity, while green bars represent lower intensity and black intermediate. The clustering of the samples is indicated by the dendrogram on the top.

The unsupervised analysis of the microarray dataset through the Principal Component Analysis (PCA, Fig. 1 panel A) showed that CALRdel52 and CALRins5-overexpressing samples clustered together and were clearly separated from CALRwt-transduced controls.

The analysis of variance (ANOVA) was subsequently performed to characterize the differentially expressed genes (DEGs) in both CALRdel52 and CALRins5 K562 cells compared to CALRwt. (Fig. 1 panel B).

As detailed above, several reports describe CALR as involved in a wide variety of signaling processes, including cellular stress responses^3,11^. Consistently with these findings, the functional analysis performed on DEGs by means of Ingenuity Pathway Analysis (IPA) showed that the categories “Unfolded Protein Response”, “Endoplasmic Reticulum Stress Pathway”, “NRF2-mediated Oxidative Stress Response” “HIF1α Signaling”, and “GADD45 Signaling” are significantly represented in the list of decreased genes in mutated vs wt K562 (Table 1**)**.

**Table 1:** Canonical Pathways significantly represented in the list of decreased genes in mutated vs wt K562 cells.

Pathway name	z-score
Unfolded protein response	−1,89
ATM Signaling	−0,816
Production of Nitric Oxide and Reactive Oxygen species	−2,449
DNA Double-Strand Break Repair by Homologous Recombination	−2,449
Role of BRCA1 in DNA Damage Response	−2,236
DNA damage	−2,236
DNA Double-Strand Break Repair by Non-Homologous End Joining	−1,633
NRF2-mediated Oxidative Stress Response	−0,816
EIF2 Signaling	−1,134
Endoplasmic Reticulum Stress Pathway	−2,449
UVB-Induced MAPK Signaling	−2,236
PTEN signaling	−2,828
Protein Ubiquitination Pathway	−1,342
HIF1α Signaling	−2,449
Chronic Myeloid Leukemia Signaling	−2,236
Hereditary Breast Cancer Signaling	−2,146
p53 Signaling	−2,447
Erythropoietin Signaling	−2
GADD45 Signaling	−2,236
FLT3 Signaling in Hematopoietic Progenitor Cells	−2,236
Myc Mediated Apoptosis Signaling	−2,236
Regulation of eIF4 and p70S6K Signaling	−2
PDGF Signaling	−2,236
Acute Myeloid Leukemia Signaling	−2,236
mTOR Signaling	−2,449
IL-6 Signaling	−2,449
Thrombin Signaling	−2,121
Gα12/13 Signaling	−2,449
VEGF Signaling	−2,236
VEGF Family Ligand-Receptor Interactions	−2
Cell cycle: G2/M DNA damage Checkpoint regulation	0,707
ERK/MAPK Signaling	−0,816
Paxillin Signaling	−2
Signaling by Rho Family GTPases	−2,449

In order to discover whether and how CALR mutations could affect UPR process and oxidative stress response after appropriate stimuli, we assessed K562 cells carrying either CALRwt, *CALR*ins5 or *CALR*del52 for their ability to respond to ER and oxidative stresses.

### CALR mutations impair ER stress response

In order to analyze the effect of CALR mutations on the response to ER stress, K562 cells were cultured in hypoxic environment (1% O2) for 24 h. Severe hypoxic exposure has been shown to cause ER stress and rapid activation of the UPR^14–16^.

**Figure 2:**
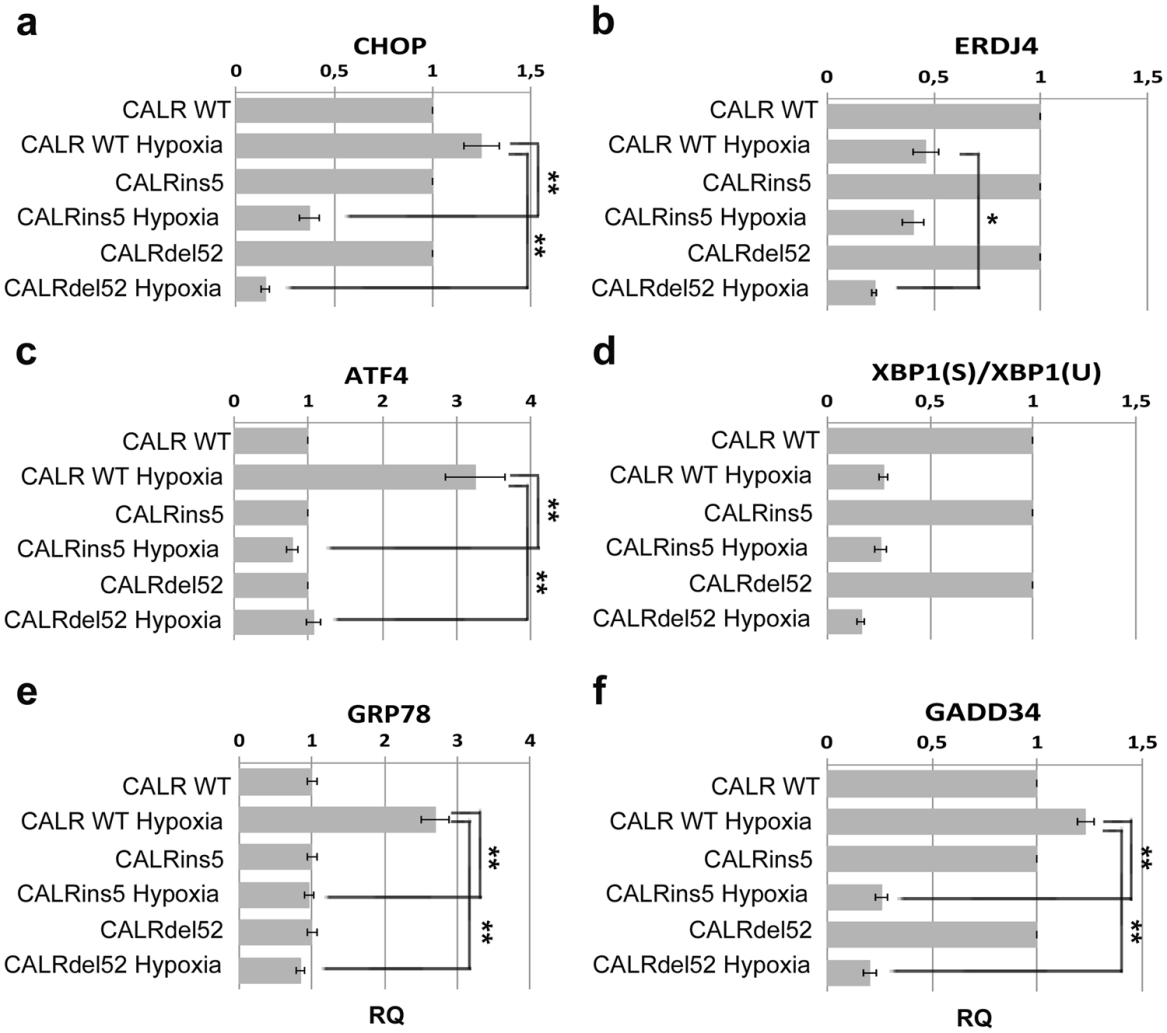
Expression of the UPR components in K562 cells expressing CALR mutated variants. Expression of the key UPR genes, CHOP (**a**), ERDJ4 (**b**), ATF4 (**c**), XBP1Spliced/XBP1Unspliced (**d**), GRP78 (**e**), and GADD34 (**f**) was measured by qRT-PCR. Results were normalized to each CALR variant sample cultured in normoxic conditions. Data are represented as Relative Quantity (RQ) mean ± S.E.M of 3 independent experiments. *p < 0.05, **p < 0.01.

Activation of the UPR response was evaluated by measuring the expression levels of the different UPR pathways components. Our results show that in case of hypoxia, in the presence of CALR mutation only PERK pathway is impaired, as demonstrated by the lower transcriptional levels of CHOP, ATF4, GRP78 and GADD34 genes in CALR-mutant K562 cells compared to CALRwt cells **(**Fig. 2, panels a,c,e,f). On the other hand, IRE1 pathway was shown to be inactive in both CALRwt and CALR-mut cells as demonstrated by the lower expression of ERDJ4 in hypoxia-cultured cells in comparison to untreated cells **(**Fig. 2, panel b). Moreover, XBP1 spliced variant, that increases during the activation of Ire1 pathway, was downregulated in response to hypoxia and did not significantly differ between CALR-mutant and CALRwt cells **(**Fig. 2, panel d).

**Figure 3:**
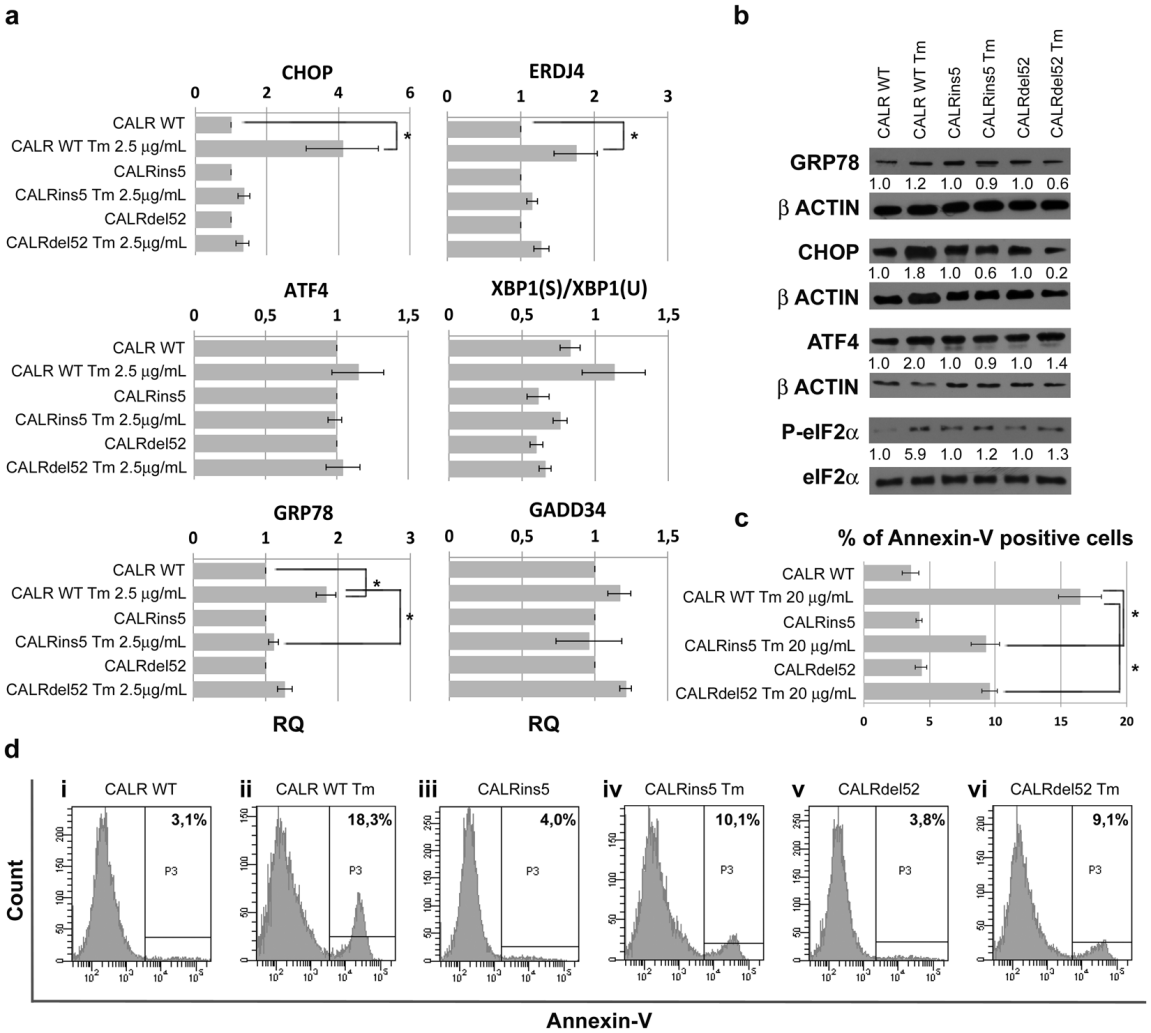
CALR mutations affect the ability to respond to ER stress. (**a**) Expression of the key UPR genes, CHOP, GRP78, ERDJ4, XBP1Spliced/XBP1Unspliced, ATF4 and GADD34 was measured by qRT-PCR after exposure to Tunicamycin (Tm) 2.5 μg/mL. Results were normalized to each untreated CALR variant sample. Data are represented as Relative Quantity (RQ) mean ± S.E.M of 3 independent experiments. (**b**) Western blot analysis of GRP78, CHOP, ATF4 and P-eIF2α protein levels in whole cell lysates from K562 cells expressing either wt or mutated *CALR* after Tm exposure. GRP78, CHOP, ATF4 and P-eIF2α protein levels in Tm treated cells were compared with the untreated sample carrying the same CALR variant. β-actin was included as loading control for GRP78, CHOP and ATF4. Total eIF2α was included as loading control for P-eIF2α. Cropped images for WB are shown, full lenght blots are presented in Supplementary Figs 2–9. (**c**) Results of Annexin V staining on K562 cells after 24 h of 20 μg/mL Tm treatment (mean ± SEM; n = 3). (**d**) Representative histograms for flow cytometry detection of Annexin V staining at 24 h after Tm treatment are shown (i: CALR WT Not Treated, ii: CALR WT Tm 20 μg/mL, iii: CALRins5 Not Treated, iv: CALRins5 Tm 20 μg/mL, v- CALRdel52 Not Treated, vi: CALRdel52 Tm 20 μg/mL). *p < 0.05.

To further assess the effects of CALR mutants on the response to ER stress, K562 cells were treated with Thapsigargin (Tg) and Tunicamycin (Tm): Tg disrupts calcium homeostasis in the ER^17^, while Tm blocks the initial step of glycoprotein biosynthesis resulting in accumulation of unfolded proteins in the ER^17^. Treatment with Tg resulted in the upregulation of the UPR genes in K562 cells expressing either wt or mutated *CALR*, with no statistically significant difference between cells carrying the wt or the mutated *CALR* variants (Supplementary Fig. 1). On the other hand, treatment with Tm confirmed the results obtained by means of hypoxia treatment. In CALR-mutated cells PERK pathway is inactive, both at the transcriptional and at the protein level **(**Fig. 3, panels a,b), compared to CALRwt K562 cells, confirming the connection between CALR mutation and the impairment of PERK response. In particular, our western blot experiments indicate that GRP78, ATF4, CHOP and the phosphorylated form of eIF2α are downregulated in CALR-mutant K562 cells compared to CALRwt cells. To investigate whether this differential activation of the UPR entails a distinct ability to respond to ER stress, K562 cells were exposed to Tm 20μg/mL for 24 h, and apoptosis was evaluated by means of Annexin V/PI staining. As expected, the deregulation of PERK pathway is reflected on the apoptosis rate induced by Tunicamycin on the different cell lines. Being PERK pathway downregulated in CALR-mutant cells, these cells exhibit a lower apoptosis rate compared to K562 CALRwt, as shown by the percentage of Annexin V-positive cells measured 24 h after Tm exposure **(**Fig. 3, panels c,d). These data suggest that CALR mutations affect cell ability to respond to ER stress, in particular cells carrying mutated *CALR* are unable to induce the expression of the pro-apoptotic components of the UPR, thus becoming resistant to ER stress-induced apoptosis.

### CALR mutations impair DNA damage repair

In order to assess whether CALR mutations are able to impact on the capacity to repair the DNA damage induced by oxidative stress, K562 cells expressing either the wt or the mutated variants of *CALR* were treated with Melittin 5 μg/mL for 24 hours^18^. Melittin (MEL) is the main constituent and principal toxin of bee venom. Recently Gajski G *et al*. demonstrated that MEL induces DNA damage including oxidative DNA damage as well as increased formation of micronuclei and nuclear buds in human peripheral blood lymphocytes^18^.

**Figure 4:**
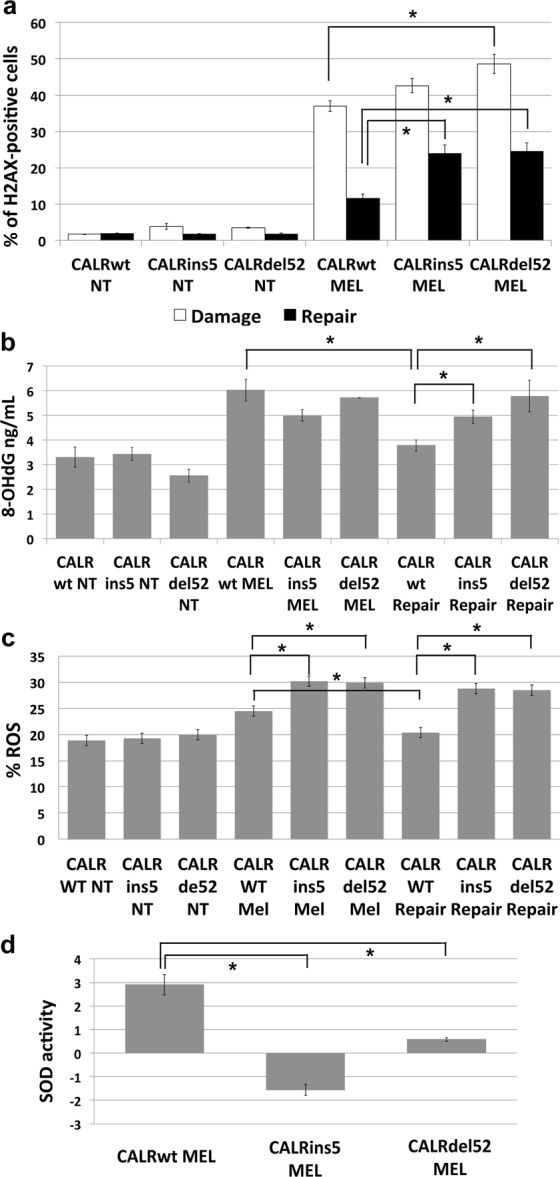
CALR mutations affect the capacity to repair oxidative stress-induced DNA damage. (**a**) Results of flow cytomeric analysis of the phosphorylation of H2AX at serine 139 (γH2AX) in K562 cells expressing either wt or mutated *CALR* after 24 h of treatment with Melittin 5 μg/mL (white bars) and after 24 h of repair (black bars) Data are reported as mean of the percentage of γH2AX-positive cells ± S.E.M of 3 independent experiments. (**b**) 8-OHdG levels measured in K562 cells expressing either wt or mutated *CALR* after 24 h of treatment with Melittin 5 μg/mL and after 24 h of repair. Data are reported as mean of 8-OHdG levels (expressed in ng/mL) ± S.E.M of 3 independent experiments. (**c**) Results of flow cytomeric analysis of ROS level in K562 cells expressing either wt or mutated *CALR* after 24 h of treatment with Melittin 5 μg/mL and after 24 h of repair. Data are reported as mean of the percentage of ROS-positive cells ± S.E.M of 3 independent experiments. (**d**) Results of SOD activity measurements. SOD activity for each sample is reported as normalized to the SOD activity of the sample collected before MEL treatment. Data are reported as mean ± S.E.M of 3 independent experiments. Abbreviations: NT, Not Treated, MEL, Melittin. *p < 0.05.

At the aim of measuring the DNA damage induced on K562 cells by MEL exposure, phosphorylation of Histone H2AX at serine 139 (γH2AX) was evaluated by means of flow cytometric analysis^19^. Our results revealed that K562 cells expressing *CALR*del52 show statistically significant higher levels of γH2AX compared to K562 cells expressing *CALR*wt (48,5% ± 2,6 vs 36,9 ± 1,4, p < 0,05) (Fig. 4, panel a); also K562 cells expressing *CALR*ins5 show higher level of γH2AX compared to *CALR*wt K562 cells (42,6% ± 2 vs 36,9 ± 1,4), even though not statistically significant. These differences are even more striking after cells are given 24 additional hours in culture to repair the damage induced by MEL exposure. After 24 h of repair, K562 cells expressing wt *CALR* were able to repair almost completely the DNA damage induced by MEL, whilst K562 cells expressing *CALR*del52 or *CALR*ins5 were not able to efficiently repair the DNA damage as evidenced by the percentage of cells positive for γH2AX (24,5% ± 2,7 vs 11,5 ± 1,8, p < 0,05; 26,1% ± 2,9 vs 11,5 ± 1,8, p < 0,05) (Fig. 4, panel a). These data clearly suggest that CALR mutations negatively impact on the capability of cells to respond to DNA damage induced by oxidative stress.

### CALR mutations impair oxidative stress response

DNA is probably the most biologically significant target of oxidative attack, among numerous types of oxidative DNA damage, the formation of 8-hydroxy-2′-deoxy-guanosine (8-OHdG) is a ubiquitous marker of oxidative stress^20^.

In order to assess whether CALR mutations are able to impact on the response to DNA damage specifically induced by oxidative stress, K562 cells expressing either the wt or the mutated variants of *CALR* were treated with Melittin 5 μg/mL for 24 hours^18^. To measure the DNA damage induced on K562 cells by MEL exposure, 8-OHdG levels were assessed by means of OxiSelect Oxidative DNA Damage ELISA Kit. Our results showed no statistically significant differences between K562 cells expressing wt *CALR* compared to *CALR* mutated variants in 8-OHdG levels after Melittin exposure (Fig. 4, panel b). On the other hand, after 24 hours of repair, K562 cells expressing wt *CALR* showed statistically significant lower levels of 8-OHdG compared to K562 cells expressing either *CALR*ins5 or *CALR*del52 (3,8 ng/mL ± 0,23 vs 4,9 ng/mL ± 0,28, 3,8 ng/mL ± 0,23 vs 5,8 ng/mL ± 0,64, respectively, p < 0,05) (Fig. 4, panel b).

The effects of *CALR*ins5 or *CALR*del52 on the response to the oxidative stress were then assessed by measuring the level of intracellular ROS by flow cytometry. *CALR*ins5 or *CALR*del52 K562 cells show statistically significant higher levels of ROS compared to *CALR*wt K562 cells after MEL treatment (30,2% ± 0,7 vs 24,5% ± 0,4; 29,9% ± 0,6 vs 24,5% ± 0,4; p < 0,05). These differences are even more remarkable after cells are left 24 additional hours in culture to reduce ROS accumulation induced by MEL exposure. In fact, after 24 h of repair, *CALR*ins5 or *CALR*del52 K562 cells were almost completely unable to reduce ROS levels induced by MEL, whilst wt K562 cells were able to efficiently counteract the ROS accumulation, as evidenced by the decreased percentage of cells positive for ROS (29,8% ± 0,6 vs 20,4% ± 0,36; 28,5% ± 0,55 vs 20,4% ± 0,36; p < 0,05) **(**Fig. 4, panel c), suggesting that CALR mutant proteins negatively affect cell ability to respond to ROS intracellular accumulation.

### CALR mutations impair Superoxide Dismutase activity

Superoxide Dismutases (SODs) catalyze the dismutation of the superoxide radical (O_2_●) into hydrogen peroxide (H_2_O_2_) and elemental oxygen (O2) which diffuses into the intermembrane space or mitochondrial matrix, and thus providing an important defense against the toxicity of superoxide radicals.

To unravel the molecular mechanism responsible for the increased oxidative stress-induced DNA damage in K562 cells carrying CALR mutations, the activity of SOD was measured by means of Superoxide Dismutase Assay Kit. To this end, K562 cells expressing either *CALR*wt, *CALR*ins5 or *CALR*del52 were treated for 24 h with MEL 5 μg/mL and SOD activity was measured before and after treatment. Our results showed an increased SOD activity in K562 cells expressing wt *CALR* after oxidative stress exposure, on the contrary, *CALR*ins5 or *CALR*del52 K562 cells demonstrated a significant lower SOD activity after MEL exposure compared to CALR*wt* K562 cells **(**Fig. 4, panel d).

### Effects of Miltirone treatment on K562 cells carrying either *CALR* wt, *CALR*ins5 or *CALR*del52

**Figure 5:**
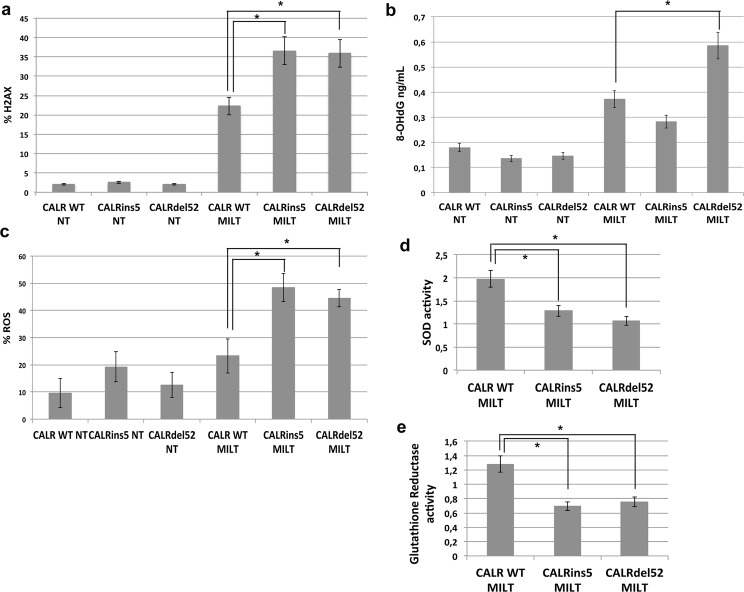
Effects of miltirone treatment on K562 cells carrying either *CALR* wt, *CALR*ins5 or *CALR*del52. (**a**) Results of flow cytomeric analysis of the phosphorylation of H2AX at serine 139 (γH2AX) in K562 cells expressing either wt or mutated *CALR* after 24 h of treatment with Miltirone 10 μM. Data are reported as mean of the percentage of γH2AX-positive cells ± S.E.M of 3 independent experiments. (**b**) 8-OHdG levels measured in K562 cells expressing either wt or mutated *CALR* after 24 h of treatment with Miltirone 10 μM. Data are reported as mean of 8-OHdG levels (expressed in ng/mL) ± S.E.M of 3 independent experiments. (**c**) Results of flow cytomeric analysis of ROS level in K562 cells expressing either wt or mutated *CALR* after 24 h of treatment with Miltirone 10 μM. Data are reported as mean of the percentage of ROS-positive cells ± S.E.M of 3 independent experiments. (**d**) Results of SOD activity measurements. SOD activity for each sample is reported as normalized to the SOD activity of the sample collected before Miltirone treatment. Data are reported as mean ± S.E.M of 3 independent experiments. (**e**) Results of GSR activity measurements. GSR activity for each sample is reported as normalized to the GSR activity of the sample collected before Miltirone treatment. Data are reported as mean ± S.E.M of 3 independent experiments. Abbreviations: NT, Not Treated, MILT, Miltirone. *p < 0.05

In order to further validate our results on oxidative stress response induced by Melittin treatment, the same experiments have been performed using Miltirone as oxidative stress inducing agent. Miltirone is a naturally occurring diterpene quinine compound isolated from Salvia miltiorrhiza. A recent work from Ling Zhou *et al*.^21^ demonstrated that Miltirone induces ROS production by inhibition of mitochondrial respiratory chain complex III. WT and mutated K562 cells were treated with Miltirone (MILT) 10 μΜ for 24 h and ROS, Glutathione reductase (GSR) activity, SOD activity, γH2AX and 8-OHdG levels were measured. Our results showed that K562 cells expressing either *CALR*ins5 or *CALR*del52 show statistically significant higher levels of γH2AX compared to K562 cells expressing *CALR*wt upon Miltirone treatment (36,6% ± 5,2 vs 22,25 ± 3,6 in *CALR*ins5vs *CALR*wt; 35,9 ± 6,1 vs 22,25 ± 3,6 in *CALR*del52 vs *CALR*wt respectively, p < 0,05) (Fig. 5, panel a). Moreover, K562 *CALR*del52 treated with Miltirone show statistically significant higher levels of 8-OHdG compared to K562 cells expressing *CALR*wt (0,59 ng/mL ± 0,02 vs 0,37 ng/mL ± 0,01, p < 0,05) (Fig. 5, panel b). In agreement with these results, ROS levels measured after Miltirone treatment showed a significant increase in both K562 carrying mutated *CALR* compared to wt K562 (48,4 ± 5,1 vs 23,2 ± 3,3 in *CALR*ins5vs *CALR*wt and 44,5 ± 3,1 vs 23,2 ± 3,3 in *CALR*del52 vs *CALR*wt) (Fig. 5, panel c).

Finally, to unravel the molecular mechanism responsible for the increased oxidative stress-induced DNA damage in K562 cells carrying CALR mutations, the activity of SOD and of GSR were measured upon Miltirone treatment. Our data revealed that both SOD and GSR activity were decreased in K562 cells carrying CALR mutated variants compared to wt K562 cells as shown in Fig. 5 panels d,e, respectively.

### OXR1 silencing impair oxidative stress response in CD34+ cells

The oxidation resistance gene 1 (OXR1) plays a critical role in protecting the cell against oxidative stress and the consequent oxidative stress-induced cell death. OXR1 stimulates the expression of antioxidant genes through the p21 pathway in order to suppress hydrogen peroxide-induced oxidative stress^22,23^.

**Figure 6:**
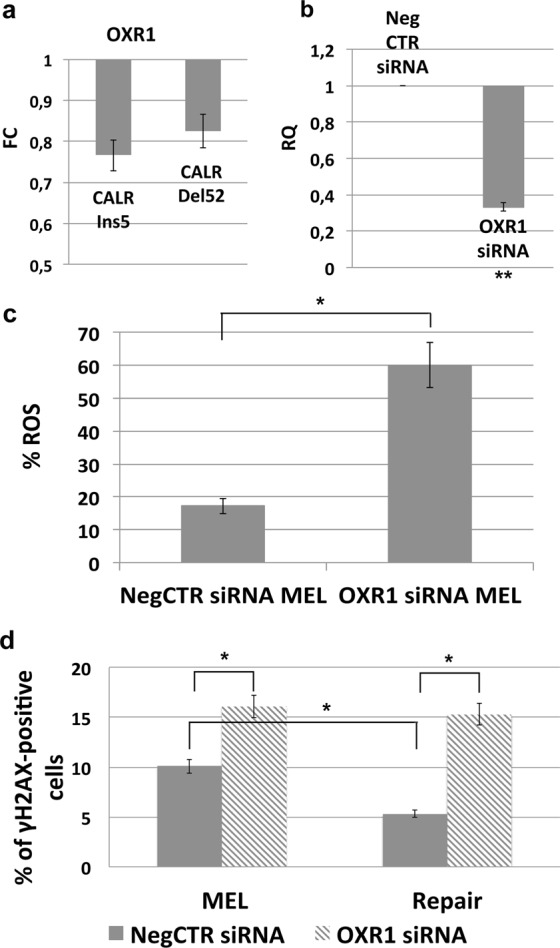
Effect of OXR1 silencing on the capacity to repair oxidative stress-induced DNA damage. (**a**) Expression level of OXR1 mRNA in the comparison K562 CALR mutated vs wt, data are reported as Fold Change (FC) ± S.E.M of 3 independent experiments. (**b**) Expression levels of OXR1 mRNA 24 hours after the last nucleofection as evaluated by qRT-PCR. Data are reported as RQ mean ± S.E.M of 3 independent experiments. (**c**) Results of flow cytomeric analysis of ROS level in CD34+ cells treated with either NTsiRNA or OXR1siRNA and Melittin 5 μg/mL. Data are reported as mean of the percentage of ROS-positive cells ± S.E.M of 3 independent experiments. (**d**) Results of flow cytomeric analysis of the phosphorylation of H2AX at serine 139 (γH2AX) in CD34+ cells treated with either NTsiRNA or OXRsiRNA after 24 h of treatment with Melittin 5 μg/mL (left) and after 24 h of repair (right). Data are reported as mean of the percentage of γH2AX-positive cells ± S.E.M of 3 independent experiments. Abbreviations: NTsiRNA, non-targeting siRNA; MEL, Melittin. *p < 0.05, **p < 0.01.

Since OXR1 mRNA is downregulated in the comparison K562 CALR mutated vs wt (Fig. 6, panel a), we wondered whether OXR1 downregulation could be a potential mechanism mediating the effect on oxidative stress response in CALR mutated cells. In this light, we performed OXR1 silencing in CD34+ cells, which are the target cells of CALR mutation and from whom the myeloproliferative disease originates.

We then assessed the effects of OXR1 knockdown on the capacity of CD34+ cells to respond to oxidative stress. OXR1 knockdown was achieved by the nucleofection of OXR1-targeting siRNA (OXR1siRNA), which was compared with a non-targeting siRNA-transfected sample (NegCTRsiRNA), as previously described^24,25^.

RealTime qRT-PCR analysis, performed 24 hours after treatment, confirmed the downregulation of mRNA levels in OXR1siRNA sample compared to control (RQ ± S.E.M, 0,3328 ± 0,02321, p < 0.01) **(**Fig. 6, panel b).

The effects of OXR1 knockdown on the response to the oxidative stress were firstly assessed by measuring the level of intracellular ROS after MEL treatment. Flow data showed a remarkable increase of ROS content in OXR1siRNA vs NegCTRsiRNA sample (17,15% ± 2,25 vs 60,2 ± 6,9, p < 0,01) **(**Fig. 6, panel c), suggesting that OXR1 silencing affects the capacity of counteract the ROS accumulation in the cell.

Next, the levels of phosphorylation of Histone H2AX at serine 139 were evaluated by flow cytometric analysis. Noteworthy, OXR1siRNA sample showed statistically significant higher levels of γH2AX compared to NegCTRsiRNA sample (16,1% ± 0,6 vs 10,1 ± 0,36, p < 0,01) (Fig. 6, panel d). These differences were even more remarkable after cells are left 24 additional hours in culture to repair the damage induced by MEL exposure. After 24 h of repair, OXR1siRNA cells were unable to repair the DNA damage induced by MEL, whilst NegCTRsiRNA cells were able to efficiently repair the DNA damage as evidenced by the percentage of cells positive for γH2AX (15,3 ± 0,4 vs 5,3% ± 0,3, p < 0,01) (Fig. 6, panel d).

These data suggest that OXR1 silencing negatively impacts on the capability of cells to respond to DNA damage, this in turn might lead to genomic instability and tendency to accumulate further mutations which is one of the main characteristics of MPNs.

## Discussion

In 2013, somatic CALR mutations were identified in most JAK2-unmutated patients with Essential Thrombocythemia (ET) or Primary Myelofibrosis (PMF) patients^4,5^. So far, 36 different types of CALR mutants have been reported in MPNs^5^. All these mutations consist of deletions and/or insertions, leading to a 1bp-frameshift thus generating an alternative reading frame causing the loss of most of the acidic C-terminal domain and of the KDEL signal.

CALR mutations are able to induce the development of an ET-like phenotype in mice, and in case of CALRdel52, also the progression to myelofibrosis^6^. Mutated CALR binds to the Thrombopoietin receptor (MPL), causing its dimerization and activation and thus leading to the constitutive activation of JAK2 signalling pathway^8,9^. In particular, Chachoua *et al*. demonstrated the necessity of MPL for CALR-mediated cellular transformation^7^. Recently Pronier *et al*. by means of affinity chromatography coupled with mass spectrometry proteomics, described the CALR-mutant interactome and demonstrated that CALR mutations promote the formation of abnormal protein chaperone complexes^26^. In particular, CALRdel52 or CALRins5 were described to bind preferentially to UPR, cytoskeletal, and ribosomal proteins. In the same work the authors demonstrated that CALRdel52 is also able to interact with the megakaryocytic transcriptional factor Fli1, which increases MPL transcription, contributing to the pathogenic phenotype.

So far, the role of mutated CALR in the development of MPNs has been strictly linked to MPL, and no data are available on the effects exerted by CALR mutations on the functions that CALR plays in the ER under homeostatic conditions.

For this reason, in order to assess whether and how CALR mutations could affect physiological CALR protein functions and thus contributing through other mechanisms to the development of MPNs, we decided to study the role of mutated CALR in K562 cells, devoid of MPL expression^12,13^. The goal of this study was to investigate whether CALR mutants could affect additional signaling pathways that might cooperate with the cellular transformation mediated by MPL activation. These pathways might have been underestimated in previous works performed on MPL-expressing cells. In particular, the observation that only 16,7% of CALR-mutated patients show a reduction in CALR allele burden after Ruxolitinib treatment^27^ suggest that the hyperactivation of the JAK2-STAT5 signaling pathway downstream MPL might not be the only pathogenetic mechanism in CALR-mediated MPN development. For these reasons K562 cells were chosen as cellular model despite their limitations as MPN disease model, such as the expression of the Bcr-Abl fusion protein.

K562 cells stably expressing either wt *CALR* or the two most common CALR mutated variants *CALR*del52 and *CALR*ins5 were generated via retroviral mediated gene transfer.

In order to identify common signaling pathways modulated by CALR mutants, GEP analysis was performed. IPA analysis performed on DEGs revealed that the categories “Unfolded protein response”, “Endoplasmic Reticulum Stress Pathway”, “NRF2-mediated Oxidative Stress Response” “HIF1α Signaling”, and “GADD45 Signaling” were significantly represented in the list of decreased genes in the comparison mutated vs wt K562 (Table 1**)**. Based on these findings, the ability to respond to ER and oxidative stresses were assessed in K562 carrying either wt or mutated *CALR*.

The Unfolded Protein Response (UPR) is the adaptation of the cell to the ER stress and it is needed to re-establish normal ER function or to initiate apoptosis^28^. The function of the UPR is performed through three distinct signalling pathways: inositol-requiring enzyme 1 alpha (IRE-1α), PKR-like ER kinase (PERK), and activating transcription factor 6 alpha (ATF6α)^29,30^. Our data showed the upregulation of the UPR target genes GRP78 and ERDJ4 (Figs 2 and 3a) and of the PERK pathway constituents CHOP, ATF4, and P-eIF2α (Fig. 3b**)** upon ER stress induction in K562 cells carrying wt *CALR*. On the contrary, K562 cells expressing either *CALR*del52 or *CALR*ins5 showed no activation of the UPR upon either hypoxia or tunicamycin exposure (Figs 2 and 3). Moreover, PI/Annexin V analysis performed after Tm treatment revealed a strong induction of apoptosis in wt K562 cells whereas *CALR*del52 and *CALR*ins5 K562 cells were less sensitive to Tm-induced apoptosis. (Fig. 3c,d). Altogether these data suggest that CALR mutations are able to affect the capability of cells to respond to ER stress, in particular they reduce the ability to activate the pro-apoptotic program of the UPR. This mechanism has been already described in solid tumors as a potential oncogenic mechanism, allowing tumor cells to grow and adapt to a hypoxic microenvironment^31^. Several evidences have revealed a dual role for the UPR in cancer. For example, PERK activation provides both pro-apoptotic and anti-apoptotic response depending on the severity of the stress. In this view, K562 cells expressing *CALR* mutated variants showed a reduced ability to induce the pro-apoptotic pathway downstream PERK, therefore CALR mutations might confer resistance to ER stress-induced apoptosis. These results are consistent with the role of chaperone that *CALR* exerts in the ER. *CALR* together with Calnexin (*CNX*) associate transiently with newly synthetize glycoproteins and promote proper folding and quality control in the ER. Previous report from Nakamura K. *et al*.^32^ showed that *CALR* overexpression induced an increased sensitivity of HeLa cells to Thapsigargin-induced apoptosis. CALR mutations cause the loss of most of the acidic C-terminal domain and the loss of the KDEL signal responsible for the retention of the protein in the ER, therefore these mutations might easily impact on *CALR* chaperone activity causing a deregulation of the UPR.

Next, we assessed whether CALR mutations could affect cell response to oxidative stress. To this end, K562 cells were exposed to Melittin 5 μg/mL, which has been previously demonstrated to induce oxidative DNA damage in human peripheral blood lymphocytes^18^ or to Miltirone 10μM, which induces ROS production by inhibition of mitochondrial respiratory chain complex III^21^. K562 cells bearing mutated *CALR* showed statistically significant higher levels of γH2AX compared to K562 cells expressing *CALR*wt after both Melittin and Miltirone treatment (Figs 4a and 5a). Moreover, K562 cells expressing *CALR*del52 or *CALR*ins5 were not able to efficiently repair the DNA damage, whilst K562 cells expressing wt *CALR* were able to repair almost completely the DNA damage induced by MEL. Accordingly, when 8-OHdG levels were measured after Melittin exposure no significant differences were noticed between K562 cells expressing wt *CALR* compared to *CALR* mutated variants, on the contrary, after 24 hours of repair, K562 cells expressing wt *CALR* showed statistically significant lower levels of 8-OHdG compared to K562 cells expressing either *CALR*ins5 or *CALR*del52 **(**Fig. 4b**)**. Overall, these data suggest that CALR mutations negatively impact on the capability of cells to respond to oxidative stress-induced DNA damage, in particular the accumulation of 8-OHdG has been shown to be a mutagenic lesion, being able to mispair with adenine thus causing G: C to T: A transversion^33^. This in turn will lead to the predisposition of cells carrying CALR mutated variants to acquire additional mutations, thus contributing the “mutator-phenotype” described for MPNs^34^.

To further understand the effects of *CALR*ins5 or *CALR*del52 on the response to oxidative stress, the level of intracellular ROS was measured. Our data showed that K562 cells carrying mutated *CALR* show higher level of intracellular ROS compared to *CALR*wt K562 after both Melittin and Miltirone exposure (Figs 4c and 5c). Moreover mutated K562 cells were unable to reduce intracellular ROS levels, whilst *CALR*wt K562 cells were capable to efficiently counteract the ROS accumulation after MEL treatment, suggesting that CALR mutants negatively affect the cell ability to fight ROS intracellular accumulation (Fig. 4c). In agreement with these findings, K562 cells carrying either *CALR*ins5 or *CALR*del52 showed decreased levels of SOD and GSR activity when exposed to oxidative stress. These results suggest that CALR mutations have a negative impact on SOD and GSR activity, therefore making cells carrying such mutations more sensible to oxidative stress. This, in turn, will lead to increased sensitivity to oxidative stress-induced damage, such as increased DNA damage and genomic instability. The importance of ROS in the development and progression of the MPN disease began to be elucidated few years ago: MPNs patients show elevated levels of ROS *in vivo*^35^ and MPN cells have been shown to produce excessive ROS *in vitro*^36^. The JAK2^V617F^ mutation is able to induce accumulation of ROS in the hematopoietic stem cell compartment: this ROS overproduction mediates the JAK2^V617F^–induced DNA damages leading to disease progression^36^. Our results suggest that also CALR mutations could impact on ROS accumulation and on the response to oxidative stress. In agreement with our findings, Ihara Y. *et al*. reported that CALR overexpression sensitizes myocardiac H9c2 cells to oxidative stress-induced apoptosis. The authors demonstrated that CALR regulates the sensitivity to apoptosis under oxidative stress through a change in Ca2+ homeostasis in myocardiac cells^10^. In this view, CALR mutations causing the loss of most of the acidic C-terminal domain responsible for the binding of Ca2+ might impact on the ability of CALR to regulate Ca2+ during oxidative stress therefore impairing the capability of cells to react to ROS.

In order to unravel the molecular mechanisms responsible for the CALR mutants-mediated increased sensitivity to oxidative stress, gene silencing experiments have been performed. Among downregulated genes in the comparison mutated-*CALR* K562 vs wt*CALR* K562, our analysis highlighted OXR1 mRNA (Fig. 6a). OXR1 acts as a sensor of cellular oxidative stress to regulate the transcriptional networks required to detoxify reactive oxygen species and modulate cell cycle and apoptosis^23^. OXR1 silencing in CB CD34+ cells induced a strong increase in intracellular ROS levels (Fig. 6c) and significant higher levels of DNA damage, measured as levels of phosphorylation of Histone H2AX at serine 139 (Fig. 6d), suggesting that OXR1 affects the capability of cells to counteract the ROS intracellular accumulation, leading to increased oxidative DNA damage. The mechanisms supporting the downregulation of OXR1 in K562 cells carrying mutated CALR needs to be further elucidated. It has been previously reported that CALR can interact with the glucocorticoid, androgen and retinoic acid receptors^37,38^ therefore influencing the ability of these receptors to bind their respective DNA responsive elements. We can only speculate that CALR mutations might impact on the protein ability to interact with transcription factors regulating the response to oxidative stress. On the other hand, it has been already reported that intracellular Ca2+ levels can regulate the expression of CFTR gene at the transcriptional level^39^, therefore CALR mutations causing the loss of the Ca2+ binding domain might induce unbalances in the intracellular Ca2+ levels affecting gene expression.

In summary, our results showed for the first time the effects exerted by CALR mutations on the physiological functions played by CALR in the ER. Our work demonstrated that CALR mutants negatively impact on the UPR: in particular, CALR mutations appear to reduce the activation of the pro-apoptotic pathway downstream the UPR, therefore allowing the accumulation of misfolded proteins in the ER and conferring resistance to ER stress-induced apoptosis. Moreover, our results showed that CALR mutations also affect the capability to respond to oxidative stress: K562 cells carrying CALR mutants showed decreased SOD and GSR activity coupled to increased ROS intracellular levels, suggesting that CALR mutants impair cell ability to counteract ROS accumulation. Moreover, cells carrying CALR mutants showed increased levels of DNA damage upon oxidative stress exposure and decreased ability to repair the oxidative DNA damage.

As a whole our work identified novel integrative mechanisms that can cooperate with MPL activation to the cellular transformation induced by CALR mutants. On one side, by affecting the ability to respond to the ER stress, CALR mutants confer resistance to ER stress mediated apoptosis. On the other side, by affecting cell sensitivity to oxidative stress and reducing the capability to respond to oxidative DNA damage, CALR mutants might lead to genomic instability and tendency to accumulate further mutations, thus supporting the theory of the “mutator-phenotype” in MPNs.

## Methods

### Ethics statement

Human CD34+ cells were purified upon donor’s informed written consent from umbilical Cord Blood (CB) samples, collected after normal deliveries, according to the institutional guidelines for discarded material (Clearance of Ethical Commitee for Human experimentation of Florence: Comitato Etico Area Vasta dell’Azienda Ospedaliero-Universitaria Careggi, approval date: April 22, 2011, approval file number # 2011/ 0014777).

### Retroviral vectors packaging

The cDNAs coding for human CALR (NM_004343) and the two commonest CALR mutated variants (CALRdel52/type I and CALRins5/type II) were synthesized (service from ORIGENE TECHNOLOGIES, Inc. USA) and cloned into retroviral vector LXIDN^25^. Packaging line for LCALRwtIDN, LCALRdel52IDN, LCALRins5IDN were generated by transfection in the ecotropic Phoenix and amphotropic GP+ envAm12 cells, as previously described^25^.

### K562 cells transduction and purification

Transduction of K562 cells was performed by four cycles of infection (one every 12 h) with viral supernatant with the addition of polybrene (8 μg/ml), and 20% FBS (SIGMA-ALDRICH) in retronectin-coated plates. Untreated 24-wells plates were coated with retronectin (10 μg/cm^2^) (TAKARA BIO INC, Japan) following the manufacturer’s protocol. Then K562 cells were seeded in 24-wells plate at 3 × 10^5^ cells/ml (1 ml/well) in fresh viral supernatant. After transduction, K562 cells were maintained in Roswell Park Memorial Institute medium (RPMI-1640; EUROCLONE) supplemented with 20% FBS for additional 36 h. Transduced K562 cells were subsequently purified by means of immunomagnetic selection (EasySep “Do-It-Yourself” Selection Kit; STEMCELL TECHNOLOGIES) using the anti-human p75-NGFR mouse monoclonal antibody (BD BIOSCIENCES). Purity of the NGFR+ cell fraction was assessed by flow cytometry after labeling with PE-conjugated anti-NGFR monoclonal antibody (MILTENYI) 48 h post-purification and was always >90%.

### K562 cells culture conditions

K562 cells were seeded at 3 × 10^5^ cells/mL in RPMI-1640 medium (EUROCLONE) supplemented with 10% FBS (SIGMA-ALDRICH). Subsequent passages were performed when cells reached 60–80% confluency. To provide hypoxic environment (1% O2), cells were cultured and treated in sealed incubators calibrated for a constant hypoxic environment: 1% O2, 94% N2 and 5% CO2, at temperature 37 °C. For physiological oxygenation or normoxia (N), cells were cultured in an incubator calibrated to 21% O2. To induce ER stress, K562 cells were seeded at 5 × 10^5^ cells/mL in RPMI-1640 supplemented with 10% FBS and exposed to Tunicamycin (Tm) (SIGMA-ALDRICH) 2.5 μg/mL for 4 h and 6 h, or to Tunicamycin 20 μg/mL for 24 h, or to Thapsigargin (Tg) (SIGMA-ALDRICH) 0.1μM or 1 μM for 4 and 6 h. To induce oxidative stress, K562 cells were seeded at 5 × 10^5^ cells/mL in RPMI-1640 supplemented with 10% FBS and exposed to Melittin 5 μg/mL (SIGMA-ALDRICH) or to Miltirone 10 μM (SANTA CRUZ BIOTECHNOLOGY) for 24 hours at 37 °C in a humidified atmosphere with 5% CO2^18^. To assess the capacity of K562 cells to repair the oxidative damage induced by MEL exposure, 24 h after treatment cells were washed twice with PBS and then seeded at 5 × 10^5^ cells/mL in fresh culture medium for additional 24 h.

### RNA extraction

Total cellular RNA was harvested from 1 × 10^5^ cells from each sample using the miRNeasy Micro RNA isolation kit (QIAGEN), according to the manufacturer’s instructions. RNA samples concentration and purity (assessed as 260/280 nm and 260/230 nm ratios) were evaluated by NanoDrop ND-1000 spectrophotometer (NANODROP TECHNOLOGIES; Wilmington, DE), while RNA integrity was assessed by using the Agilent 2100 Bioanalyzer (AGILENT TECHNOLOGIES; Waldbrunn, Germany).

### Quantitative reverse transcription polymerase chain reaction (qRT-PCR)

Total RNA (100 ng) was reverse-transcribed to cDNA using a High Capacity cDNA Archive Kit (LIFE TECHNOLOGIES; Carlsbad, CA, USA). TaqMan PCR was carried out using the TaqMan Fast Advanced PCR master mix and TaqMan gene expression assays (all reagents from LIFE TECHNOLOGIES), by means of a 7900HT Fast Real-Time PCR System (APPLIED BIOSYSTEMS) as previously reported^40^.

### DNA damage measurement

The phosphorylation of Histone H2AX at serine 139 (γH2AX) was evaluated by means of flow cytometric analysis. Briefly, 1 × 10^5^ cells were fixed and permeabilized with Cell Signaling Buffer Set A (MILTENYI BIOTEC GmbH) and then stained with the Anti-H2AX pS139-FITC, (clone: REA502, MILTENYI BIOTEC GmbH) for 30 minutes in the dark at room temperature. After staining, cells were analyzed by using a BD FACSCanto II (BD BIOSCIENCES; San Jose, CA USA). At least 10,000 events were counted for each sample to ensure statistical relevance.

### Annexin V/PI staining

Apoptosis was evaluated by Annexin V assay (Annexin V-FITC Kit, TREVIGEN INC.) following manufacturer protocol. Briefly, 5 × 10^5^ cells were washed with cold PBS and incubated in 100 μL Annexin V Incubation Reagent for 15 min at room temperature in the dark. After staining, cells were analyzed by using a BD FACSCanto II (BD BIOSCIENCES; San Jose, CA USA). At least 10,000 events were counted for each sample to ensure statistical relevance.

### 8-OHdG assay

Genomic DNA was extracted from K652 cells carrying either *CALR*wt, *CALR*ins5 or *CALR*del52 after treatment with MEL 5 μg/mL for 24 h, after 24 h of repair or after treatment with Miltirone 10 μM for 24 h by means of DNeasy Blood and Tissue kit (QIAGEN). Genomic DNA was extracted from treated/untreated cells following a standard molecular biology protocol and re-suspended in 100 μL water. The same amount of genomic DNA (3 μg) was used for the detection of 8-OH-dG level by means of the OxiSelect Oxidative DNA Damage ELISA Kit (CELL BIOLABS, San Diego, California, USA), following the manufacture’s instruction.

### Detection of ROS intracellular level

The redox-sensitive fluorochrome 5-(and 6)-chloromethyl-2′,7′-dichlorodihydroflurescein diacetate dye (CM-H_2_DCFDA, INVITROGEN) was used to measure the intracellular Reactive Oxygen Species (ROS). Briefly, K562 or CD34+ cells were loaded with 2 μM 5-(and6)-chloromethyl-2′, 7′-dichlorodihydroflurescein diacetate for 20 min at 37 °C. Before analysis by flow cytometry, the cells were removed from loading buffer and incubated in growth medium for 1 h at 37 °C^41^. Data acquisition and analysis was performed using a BD FACSCanto II (BD BIOSCIENCES; San Jose, CA USA). At least 10,000 events were detected for each sample to guarantee the statistical significance.

### Measurement of SOD activity

SOD activity was measured by means of Superoxide Dismutase Assay Kit (TREVIGEN, INC. Catalog # 7500-100-K) following manufacturer instruction. Briefly, 1 × 10^6^ K562 cells expressing either *CALR*wt, *CALR*ins5 or *CALR*del52 were treated for 24 h with MEL 5 μg/mL or Miltirone 10 μM and then lysed in 5 volumes cold 1X Cell Lysis Solution. 15 μg of protein/sample were used to perform the assay.

### Measurement of GSR activity

GSR activity was measured by means of Glutathione Reductase Assay Kit (TREVIGEN, INC. Catalog # 7510-100-K) following manufacturer instruction. Briefly, 1 × 10^6 K562 cells expressing either *CALR*wt, *CALR*ins5 or *CALR*del52 were treated for 24 h with Miltirone 10 μM and then lysed in 5 volumes cold 1 X Tissue Homogenization. 15 μg of protein/sample were used to perform the assay.

### Human CD34+ Hematopoietic Stem/Progenitor Cells (HSPCs) purification

Cord Blood (CB) CD34+ cells were purified as previously described^42^. After immunomagnetic separation, CD34+ cells were seeded in 24-well plates at 5 × 10^5^/ml in Iscove’s modified Dulbecco’s medium (IMDM) (GIBCO, Grand Island, NY, USA) containing 20% Human Serum (BIO-WHITTAKER, Walkersville, MD, USA), SCF (50 ng/ml), Flt3-ligand (Flt3L) (50 ng/ml), TPO (20 ng/ml), IL-6 (10 ng/ml) and IL-3 (10 ng/ml) (all from MILTENYI BIOTEC).

### OXR1 silencing in CD34+ cells

Human CD34+ cells were transfected by using the 4D-Nucleofector System (LONZA) as previously reported^43^. Briefly, starting from the day after CD34+ cell purification, each sample was electroporated three times, once every 24 hours, with a small interfering RNA (siRNA) targeting human OXR1 mRNA (LIFE TECHNOLOGIES, siRNA ID s115). To exclude non-specific effects caused by interfering RNA (RNAi) nucleofection, a sample transfected with a nontargeting siRNA (NTsiRNA; LIFE TECHNOLOGIES) was included. Cells were analyzed 48 h after the last nucleofection for both cell viability and OXR1 mRNA expression.

### Statistical analysis

The statistic used for data analysis was based 2-tailed Student *t*-tests for average comparisons in paired samples (equal variance). Data were analyzed with Microsoft Excel (MICROSOFT OFFICE, 2011 release) and are reported as mean ± standard error of the mean (SEM). A p-value < 0.05 was considered significant.

## Supplementary information

Supplementary material

## Data Availability

The datasets generated during the current study are available in the Gene Expression Omnibus repository (http://www.ncbi.nlm.nih.gov/geo), series # GSE114414 and GSE127250. GEP data can be downloaded at the link https://www.ncbi.nlm.nih.gov/geo/query/acc.cgi?acc = GSE114414 and https://www.ncbi.nlm.nih.gov/geo/query/acc.cgi?acc = GSE127250.
