# Supplementary material for: Calreticulin Ins5 and Del52 mutations impair unfolded protein and oxidative stress responses in K562 cells expressing CALR mutants
Source: Sci Rep. 2019 Jul 22;9:10558. doi: 10.1038/s41598-019-46843-z (PMC6646313; doi:10.1038/s41598-019-46843-z)
Supplement: Supplementary file 1 — Supplementary material [file 41598_2019_46843_MOESM1_ESM.pdf]

Calreticulin Ins5 and Del52 mutations impair unfolded protein and oxidative stress responses in K562 cells expressing CALR mutants.

Simona Salati [1], Elena Genovese [1], Chiara Carretta [1], Roberta Zini [1], Niccolò Bartalucci [3], Zelia Prudente [1] ,Valentina Pennucci [2], Samantha Ruberti [1], Chiara Rossi [1], Sebastiano Rontauroli [1], Elena Enzo [1], Laura Calabresi [3], Manjola Balliu [3], Carmela Mannarelli [3], Elisa Bianchi [1], Paola Guglielmelli [3], Enrico Tagliafico [4], Alessandro M. Vannucchi [3] and Rossella Manfredini [1]

1 Centre for Regenerative Medicine, University of Modena and Reggio Emilia, Modena, Italy. 2 Institute for Cell and Gene Therapy & Center for Chronic Immunodeficiency, University of Freiburg, Germany. 3 CRIMM, Center for Research and Innovation for Myeloproliferative Neoplasms, Department of Experimental and Clinical Medicine, AOU Careggi, University of Florence. 4. Center for Genome Research, University of Modena and Reggio Emilia, Modena, Italy.

¶Corresponding authors: Rossella Manfredini, PhD, Centre for Regenerative Medicine "Stefano Ferrari", University of Modena and Reggio Emilia, via Gottardi n.100, 41125 Modena, Italy. E-mail: rossella.manfredini@unimore.it.

Gene Expression Profiling (GEP)

GEP was performed on RNA samples isolated from wt, *CALR*del52 and *CALR*ins5 K562 cells immediately after NGFR-based sorting from 3 independent experiments. As far as GEP upon Melittin exposure is concerned, RNA samples were isolated from wt, *CALR*del52 and *CALR*ins5 K562 after 24h of Melittin 5μg/mL treatment.

GEP cDNA synthesis and biotin-labeled target synthesis were performed using the GeneAtlas 3´ IVT Express Kit according to the protocol supplied by Affymetrix. The HG-U219 Array Strips (AFFYMETRIX; Santa Clara, CA) hybridization, staining and scanning were performed by using the GeneAtlas Platform.

The probe signal data were normalized and converted into expression values using the robust multiarray average (RMA) procedure. Quality control was assessed by using QC procedures included in the Partek GS. 6.6 Software Package (http://www.partek.com).

An exploratory principal component analysis (PCA) was performed by means of the PCA module implemented in Partek GS. Differentially expressed genes (DEGs) were selected on robust multiarray average (RMA)-normalized data through a supervised analysis using the ANOVA module supplied by the Partek GS. 6.6 Software Package (http://www.partek.com). Ingenuity Pathway Analysis software (IPA, version 8.6; INGENUITY SYSTEMS; Redwood City, CA, http://www.ingenuity.com) was used to predict the functional effects of CALR wt, CALRdel52 and CALRins5 overexpression based on the list of DEGs. By using the DEGs dataset as input, through the downstream effects algorithm IPA software returns the overrepresented/underrepresented biological functions

Western Blot analysis

GRP78, CHOP, ATF4, eIF2α and P-eIF2α protein levels were assessed by means of Western Blot Analysis in K562 cells carrying either CALRwt, CALRins5 or CALRdel52. Briefly, cells were harvested 4 and 6 hours after treatment with Tunicamycin 2.5 μg/mL (SIGMA ALDRICH, #SML1287-1ML), washed twice with cold phosphate-buffered saline (PBS) and lysed in 50 mM Tris (tris(hydroxymethyl) aminomethane)-Cl (pH 7.4), 150 mM NaCl, 1% Nonidet P-40, 10 mM KCl, 1 mM EDTA, 20 mM NaF, 0.25% Na doexycholate, 5 mM dithiothreitol (DTT). Protease inhibitors (Roche, #1697498) and phosphatase inhibitors (THERMOFISHER SCIENTIFIC, #1862495) were added to the lysis buffer. Total cellular lysates were loaded and separated on 10% SDS-polyacrylamide gel and then transferred on a nitrocellulose membrane (BIORAD, #1620115). Membranes were then pre-blocked in a blocking solution of 0.1% TBST containing 5% non-fat dry milk (NFDM) for GRP78,

CHOP and ATF4, in a blocking solution of 0.1% TBST containing bovine serum albumin (BSA) (2.5% for eIF2α, 5% for P-eIF2α). Then membranes were incubated with the following primary antibodies: mouse monoclonal anti-GRP78 antibody (SANTA CRUZ BIOTECHNOLOGY, #sc-376768, 1:500 dilution at 4°C overnight), mouse monoclonal anti-CHOP antibody (SANTA CRUZ BIOTECHNOLOGY, #sc-7351, 1:100 dilution at 4°C overnight), mouse monoclonal anti-ATF4 antibody (SANTA CRUZ BIOTECHNOLOGY, #sc-390063, 1:50 dilution at 4°C overnight), rabbit polyclonal anti- eIF2α antibody (CELL SIGNALING TECHNOLOGY, #9722, 1:200 dilution at 4°C overnight), rabbit polyclonal anti-Phospho-eIF2α antibody (CELL SIGNALING TECHNOLOGY, #9721, 1:200 dilution at 4°C overnight) and with rabbit polyclonal anti-β-actin primary antibody (THERMO FISHER SCIENTIFIC, #PA1-16889; 1:2000 dilution for 1 hour at RT). The blots were washed for three times with 0.1% TBST and then incubated with 1:1000 dilution of HRP-conjugated goat anti-rabbit secondary antibody (THERMO FISHER SCIENTIFIC, #32460) or with 1:2000 dilution HRP-conjugated donkey anti-mouse secondary antibody (SANTA CRUZ BIOTECHNOLOGY, #sc-2314) for 1h at RT secondary antibodies. After three successive washes with 0.1% TBST, SuperSignal West Pico Plus Chemiluminescent Substrate (THERMO SCIENTIFIC, #34577) was used for protein detection. Blot images have been processed by means of ImageJ software for protein quantification. The quantification reflects the relative amounts as a ratio of each protein band relative to the lane's loading control.

Immunofluorescence staining

Cytospins were fixed with methanol and permeabilized using 0.2% Triton X-100 in PBS for 10 minutes at room temperature. After blocking with 5% FBS and 2% BSA in PBS for 1 hour at room temperature, slides were incubated with rabbit polyclonal anti-mutated CALR (1:50 in blocking solution, kindly provided by Vannucchi AM.) or rabbit polyclonal anti-human CALR (1:50 in blocking solution, ABCAM ab39897) 1h at room temperature. This was followed by incubation with Alexafluor 568 donkey anti-rabbit secondary antibody (1:500 in blocking solution, THERMOFISHER) for 1h at room temperature. All incubations were followed by 3 washes with PBS solution. Nuclear counterstaining was performed with 4',6-diamino-2-phenylindole (DAPI). The slides were mounted with DakoCytomation fluorescent mounting medium (DAKO). Finally, fluorescence imaging was performed using the Zeiss LSM 510 Meta Confocal Microscope (ZEISS, Germany) and digital images of

representative areas were taken. To ensure random sampling, 50 images/slide were captured and cells positive for CALR were scored.

Supplementary Figure 1

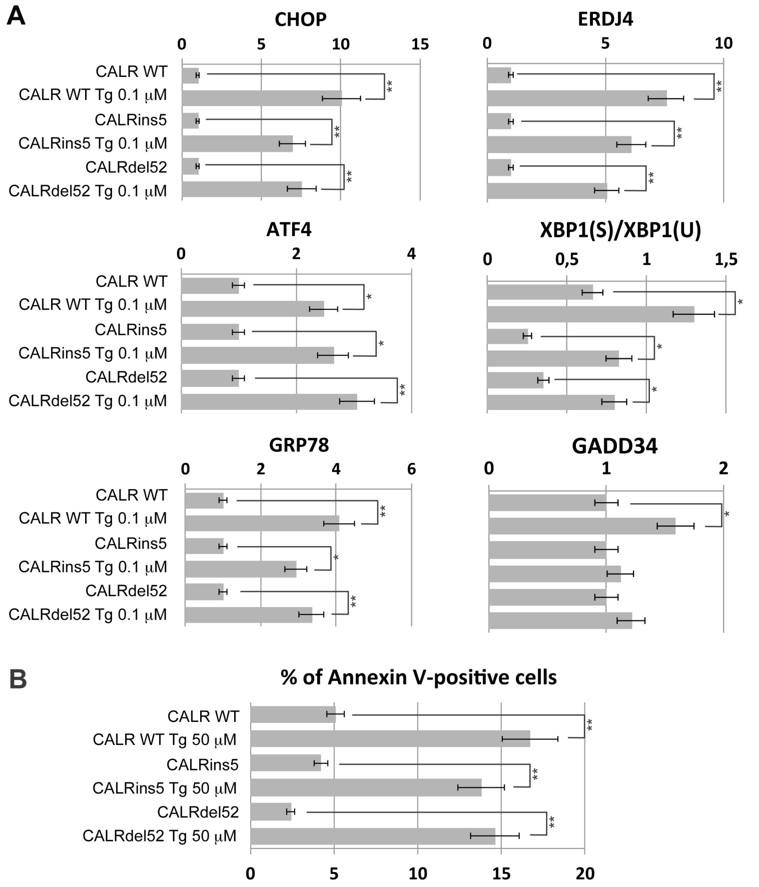

Supplementary Figure 1. CALR mutations do not affect the ability to respond to the ER stress inducer Thapsigargin. (A) Expression of the key UPR genes, CHOP, GRP78, ERDJ4, XBP1Spliced/XBP1Unspliced, ATF4 and GADD34 was measured by qRT-PCR after exposure to Thapsigargin (Tg) 0.1 μM. Results were normalized to each untreated CALR variant sample. Data are represented as Relative Quantity (RQ) mean ± S.E.M of 3 independent experiments. (B) Results of Annexin V staining on K562 cells after 24h of 50 μM Tg treatment (mean±SEM; n=3). *p<0.05, **p<0.01

Supplementary Figure 2

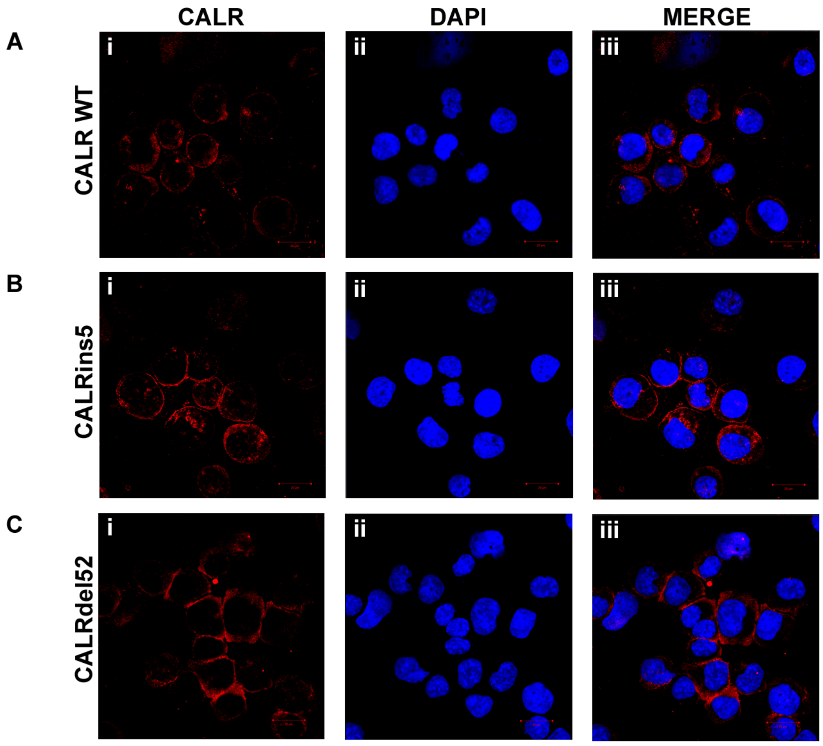

Figure 2S

Supplementary Figure 2. Subcellular localization of wild-type and mutant CALR. Confocal immunofluorescence microscopy was performed in K562 cells carrying either wt or mutated CALR and stained with anti-CALR, and 4,6 diamidino-2-phenylindole (DAPI). (a) *CALR*wt K562 cells, (b) *CALR*ins5 K562 cells and (c) *CALR*del52 K562 cells.

Supplementary Figure 3

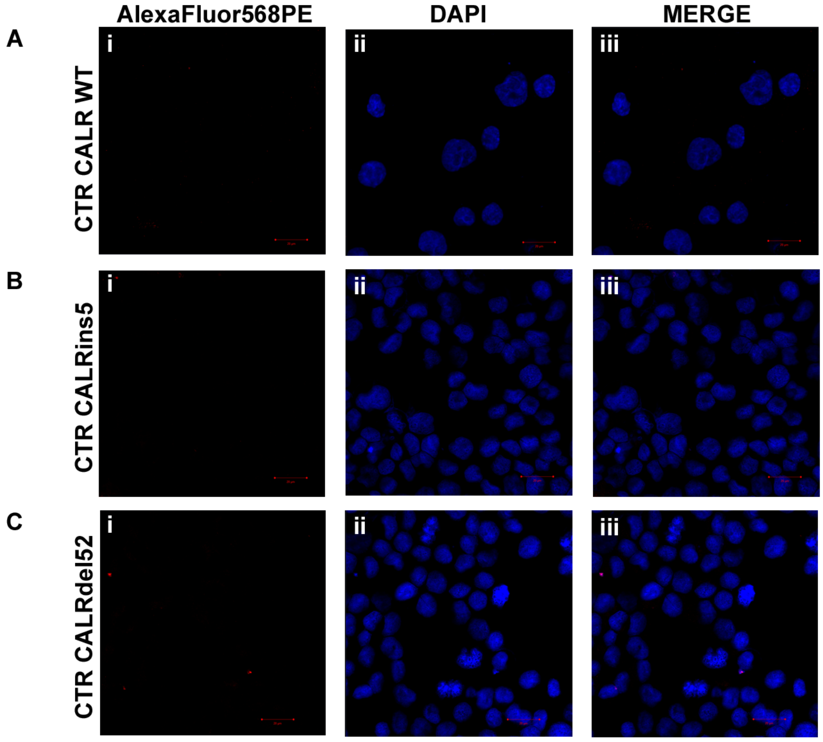

Figure 3S

Supplementary Figure 3. Control slides for subcellular localization of wild-type and mutant CALR.

Confocal immunofluorescence microscopy was performed in K562 cells carrying either wt or mutated CALR and stained with Alexafluor 568 donkey anti-rabbit IgG, and 4,6 diamidino-2-phenylindole (DAPI). (a) *CALR*wt K562 cells, (b) *CALR*ins5 K562 cells and (c) *CALR*del52 K562 cells.

Supplementary Figure 4

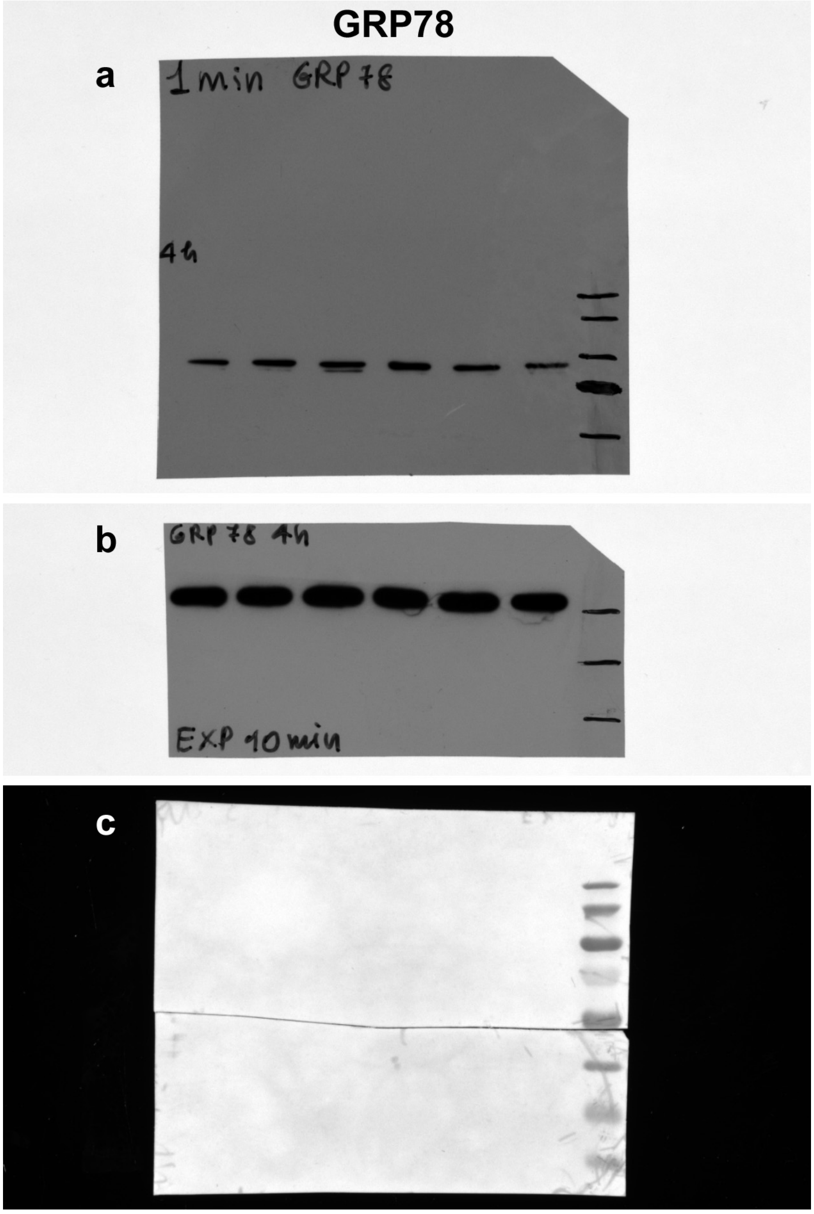

Supplementary Figure 4. Full blot image for the western blot analysis of GRP78 protein levels presented in Figure 3b. a. WB blot analysis of GRP78. b. WB analysis of Actin β protein levels used as control for GRP78. From the left: CALRdel52 Tm, CALRdel52, CALRins5 Tm, CALRins5, CALR WT Tm, CALR WT, PageRule Prestained Protein Ladder, 10 to 180 kDa (THERMOFISHER). c. full blot image, the blot was cut to perform the incubations with anti-GRP78 antibody and anti-Actin β antibody on the same membrane.

Supplementary Figure 5

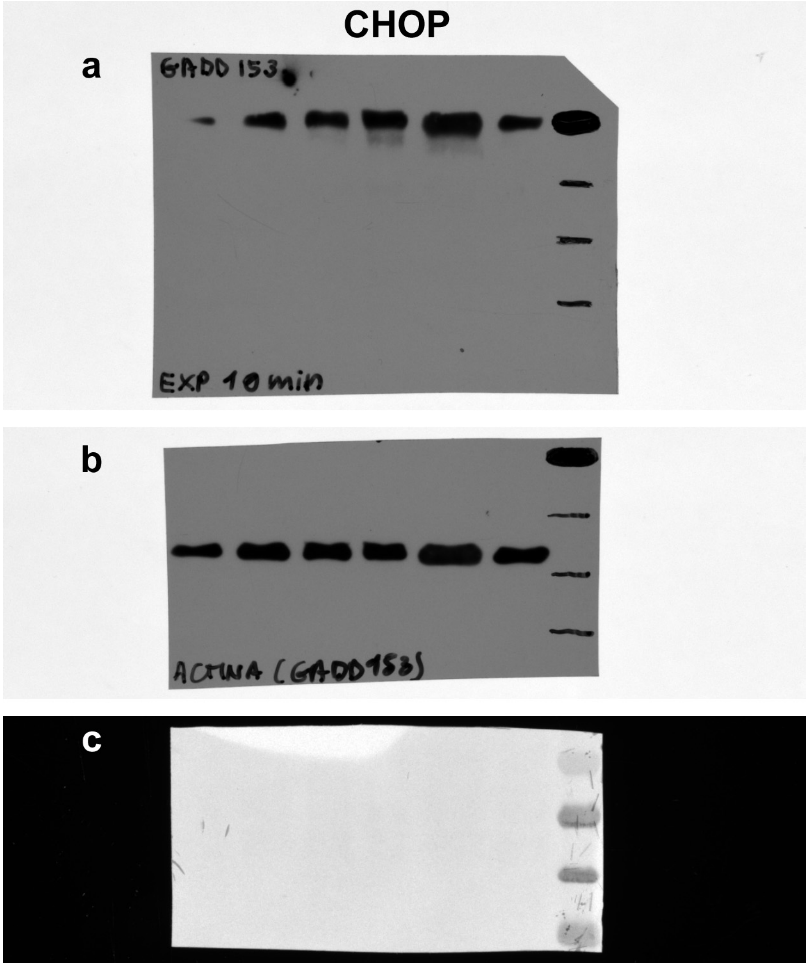

Supplementary Figure 5. Full blot image for the western blot analysis of CHOP protein levels presented in Figure 3b. a. WB blot analysis of CHOP. b. WB analysis of Actin β protein levels used as control for CHOP. From the left: CALRdel52 Tm, CALRdel52, CALRins5 Tm, CALRins5, CALR WT Tm, CALR WT, PageRule Prestained Protein Ladder, 10 to 180 kDa (THERMOFISHER). c. full blot image, the blot was first incubated with anti-CHOP antibody, then stripped and incubated with anti-Actin β antibody.

Supplementary Figure 6

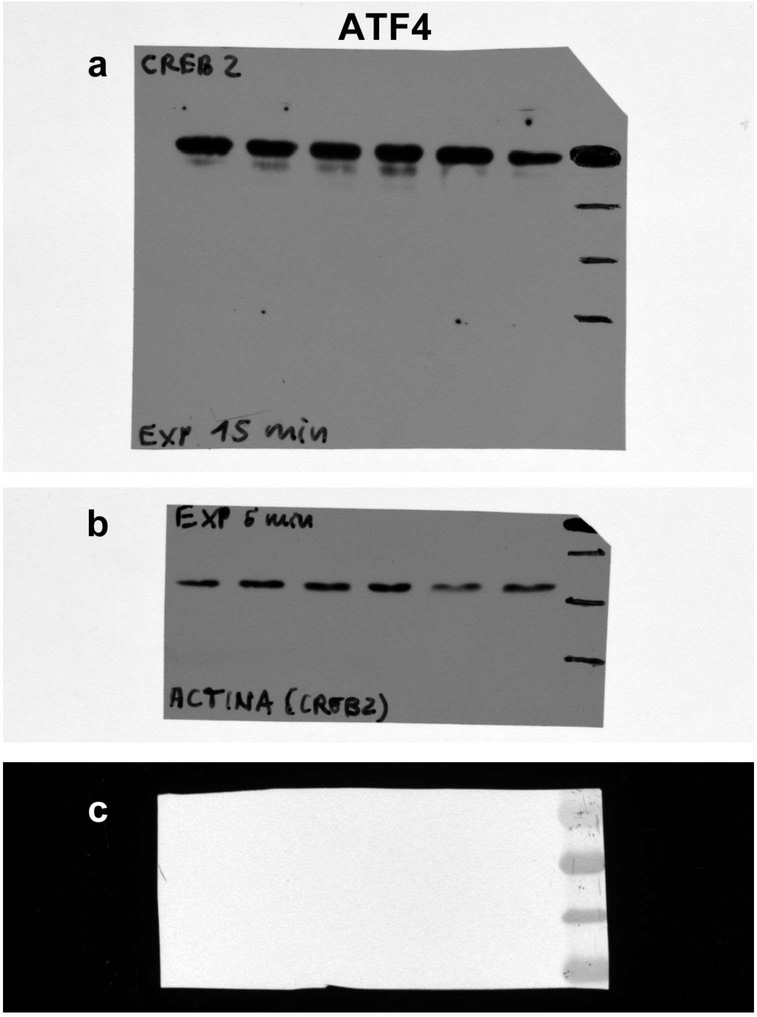

Supplementary Figure 6. Full blot image for the western blot analysis of ATF4 protein levels presented in Figure 3b. a. WB blot analysis of ATF4. b. WB analysis of Actin β protein levels used as control for ATF4. From the left: CALRdel52 Tm, CALRdel52, CALRins5 Tm, CALRins5, CALR WT Tm, CALR WT, PageRule Prestained Protein Ladder, 10 to 180 kDa (THERMOFISHER). c. full blot image, the blot was first incubated with anti-ATF4 antibody, then stripped and incubated with anti-Actin β antibody.

Supplementary Figure 7

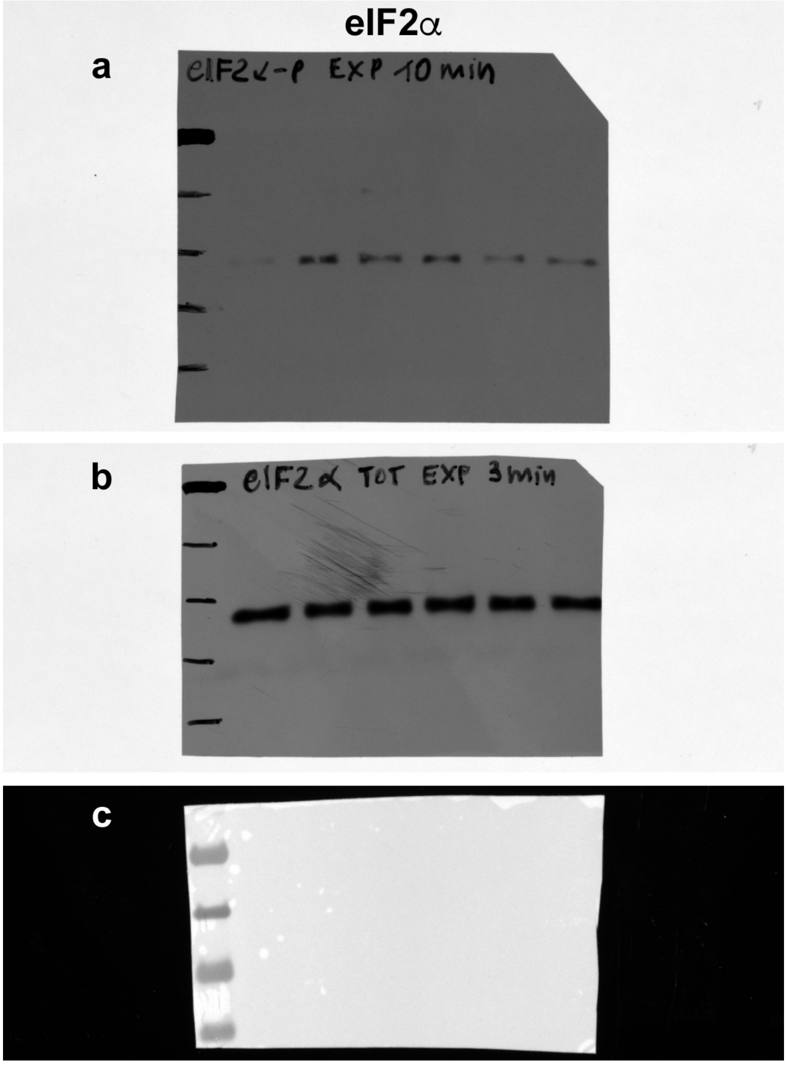

Supplementary Figure 7. Full blot image for the western blot analysis of P-eIF2α protein levels presented in Figure 3b. a. WB blot analysis of P-eIF2α. b. WB analysis of total eIF2α protein levels used as control for P-eIF2α. From the left: PageRule Prestained Protein Ladder, 10 to 180 kDa (THERMOFISHER), CALR WT, CALR WT Tm, CALRins5, CALRins5 Tm, CALRdel52, CALRdel52 Tm. c. full blot image, the blot was first incubated with anti-P-eIF2α antibody, then stripped and incubated with anti-eIF2α antibody.
